# Prognostic Potential of Baseline Eosinophils at the Initiation of Immune Checkpoint Inhibitor Treatment of Metastatic Melanoma: A Systematic Review and Meta-Analysis

**DOI:** 10.1155/jskc/2561307

**Published:** 2025-11-30

**Authors:** Thilo Gambichler, Jan Overbeck, Nessr Abu Rached, Laura Susok, Sera S. Weyer-Fahlbusch

**Affiliations:** ^1^Department of Dermatology, Dortmund Hospital gGmbH, University Witten/Herdecke, Dortmund, Germany; ^2^Department of Dermatology, Ruhr-University Bochum, Bochum, Germany; ^3^Department of Dermatology, Christian Hospital Unna, Unna, Germany

**Keywords:** eosinopenia, eosinophilia, eosinophilic cationic protein, immunotherapy

## Abstract

Cutaneous melanoma (CM) remains a rapidly rising malignancy, with metastatic disease still carrying a limited 5-year survival. Immune checkpoint inhibitors (ICIs) have changed the treatment landscape, yet only a fraction of patients achieves durable benefit. Easily obtainable blood-based biomarkers are, therefore, needed to guide patient selection and prognostication. Eosinophilic granulocytes have emerged as potential modulators of antitumor immunity. In this systematic review and meta-analysis, we adhered to PRISMA guidelines and searched PubMed, Cochrane Library, and Scopus for studies published from 2011 onward that evaluated baseline peripheral eosinophil counts (absolute or percentage) as predictors of overall survival (OS) in advanced (stage III/IV) melanoma patients treated with ICI. Six cohort studies totaling 4.243 patients met inclusion criteria, each reporting hazard ratios (HRs) and 95% confidence intervals (CIs) in multivariable analyses for high versus low baseline eosinophil groups. Study quality was high (Newcastle–Ottawa Scale ≥ 7 stars), though selection biases were noted across all cohorts. A random-effects meta-analysis (DerSimonian–Laird method) initially yielded a pooled HR of 0.69 (95% CI: 0.36–1.30) for high versus low eosinophil counts, but heterogeneity was substantial (*I*^2^ = 77.8%, *p* < 0.001). Through Baujat and leave-one-out diagnostics, one study was identified as an influential outlier; its exclusion reduced heterogeneity down to 33.3% (*p*=0.20). Reanalysis of the remaining five studies (*n* = 4.158) demonstrated a significant survival advantage for patients with elevated baseline eosinophils (HR: 0.51 and 95% CI: 0.39–0.66; *p* < 0.001). Funnel plot symmetry and a nonsignificant Egger's test (*p*=0.27) indicated no evidence of publication bias. These findings support baseline peripheral eosinophil count as a cost-effective, readily available biomarker associated with improved OS under ICI therapy in advanced melanoma. However, prospective studies with standardized eosinophil thresholds, comprehensive covariate adjustment, and integrated mechanistic analyses are warranted to validate and operationalize this marker for patient stratification in clinical practice.

## 1. Introduction

Cutaneous melanoma (CM) remains one of the deadliest cancers worldwide, with incidence rates continuing to climb over the past decades. Early-stage disease can often be cured surgically, but once metastasis occurs, prognosis is poor and 5-year survival drops significantly. Traditional serum biomarkers, such as lactate dehydrogenase and S100B, offer some prognostic and diagnostic information but lack sufficient sensitivity and specificity to guide individualized therapy in advanced disease [1].

With the advent of immune checkpoint inhibitors (ICIs) targeting CTLA-4 and PD-1/PD-L1 pathways, durable responses in metastatic CM have transformed the therapeutic landscape. However, only a subset of patients derives long-term benefit, and treatment can incur severe immune-related toxicities [2]. This has fueled an urgent need for biomarkers that predict both efficacy and adverse events, ideally obtainable from routine blood tests. Eosinophilic granulocytes, which are classically involved in parasitic defense and allergic inflammation, have emerged as intriguing players in tumor immunity. Clinically, elevated peripheral or intratumoral eosinophil counts have been associated with improved progression-free survival (PFS) and overall survival (OS) in CM patients treated with ICI, as well as higher rates of immune-related adverse events, suggesting a dual role in both antitumor efficacy and inflammatory toxicity [3, 4].

Given their accessibility via standard full blood counts and plausible mechanistic links to T cell–mediated antitumor immunity, eosinophils represent a promising, cost-effective biomarker to refine patient selection and monitor response to ICI therapy. The recent systematic review by Brand et al. [1–4] synthesizes the existing clinical evidence highlighting both the potential and the current limitations of eosinophil enumeration in CM. Brand et al. [1] reported that while 28 of 33 studies suggested some prognostic role for eosinophils (outcomes, response rates, or adverse events), only a subset actually demonstrated that increases in eosinophil levels correlate with improved OS. Notably, a few reported no association, and at least two found an inverse prognostic relationship [1]. Brand et al.'s [1] recent systematic review collated 33 such studies but highlighted extreme heterogeneity: some cohorts included unresectable versus adjuvant patients, eosinophil measurements were taken from peripheral blood or tumor tissue, time points ranged from pretreatment to dynamic on-therapy sampling, and several analyses relied on derived ratios rather than raw eosinophil counts [4–22]. Likely given the great heterogeneity of study data, Brand et al. [1] could not apply meta-analysis statistics [1].

Because these varied designs can obscure the true prognostic value of eosinophils, we aimed to choose in present systematic review and meta-analysis to focus on baseline peripheral eosinophil levels (absolute counts or percentages) and their relationship with OS, thereby isolating the most clinically actionable signal from a diverse evidence base.

## 2. Materials and Methods

### 2.1. Protocol

**Figure 1:**
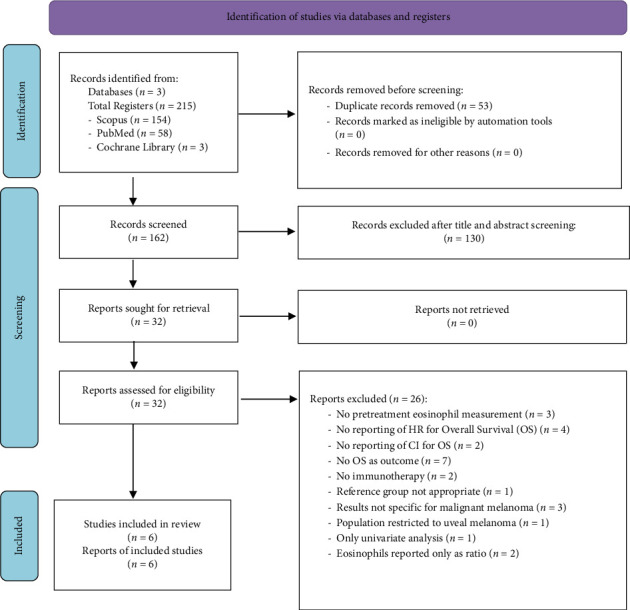
PRISMA flowchart of the present systematic review and meta-analysis.

This systematic review and meta-analysis were conducted and reported in compliance with the Preferred Reporting Items for Systematic Reviews and Meta-Analyses (PRISMA) guidelines (Figure 1 and Table 1 supporting). It is registered with the Open Science Framework https://doi.org/10.17605/OSF.IO/FTKVD, accessed on 24 April 2025 [23].

### 2.2. Search Strategies

A broad and sensitive search was carried out, with no restrictions on languages but period of publication starting from 2010 (era of novel immunotherapy era). The following databases were searched: PubMed, Cochrane Library, and Scopus. Search strategies applied are detailed in Table 2 supporting.

### 2.3. Selection of Studies

Following identification of potential studies, two independent reviewers (T. G. and J. O.) selected the included articles in three phases. In screening Phase 1, the reviewers evaluated the titles and abstracts based on the eligibility criteria. In Phase 2, the reviewers reviewed the full texts and selected the articles according to the same criteria as in screening Phase 1. Subsequently, the reviewers verified all the information obtained concerning critical inclusion and exclusion criteria. In cases of disagreement, a third reviewer (S. S. W-F. or N. A. R.) was consulted. Inclusion criteria were as follows: studies including at least 30 patients with advanced (stage III/IV) CM, novel immunotherapy (ICIs), and hazard ratios (HRs) including the 95% confidence intervals (CIs) for OS. We only included studies reporting baseline eosinophils as independent variable (absolute and/or relative) predicting OS in multivariable analyses. Data of PFS were provided only in two studies, otherwise fulfilling our inclusion criteria (Table 3 supporting). Hence, we did not perform meta-analysis of PFS data. Abstracts, posters, reviews, case reports, and patients with uveal melanoma were excluded from further analysis.

### 2.4. Data Collected

Data were collected on the characteristics of the studies, including authorship, study design, and year of publication and country, intervention modality, results, and conclusion. The primary outcome was the assessment of OS defined as the time from initiation of first immunotherapy for metastatic melanoma to death or last date of follow-up.

### 2.5. Individual Assessment of Study Quality

The Newcastle–Ottawa Scale (NOS) is a widely used tool for evaluating the quality of nonrandomized studies, such as cohort and case-control studies, included in systematic reviews and meta-analyses. The NOS assesses the quality of a study based on three main aspects: selection of study groups, comparability of the groups, and assessment of the outcomes or exposures [24].

### 2.6. Data Synthesis and Statistical Analysis

A meta-analysis was conducted to evaluate the differences between OS of patients with high or low eosinophils. HRs with corresponding 95% CI, number of patients per cohort, and treatments were extracted from each included study. All statistical analyses were performed using R and RStudio (Version 4.4.3). A forest plot was generated to visually represent the individual study estimates and the pooled random effect sizes for OS. The presence of statistical heterogeneity among studies was assessed using the *I*^2^ statistic (Cochran's Q test). *I*^2^ of 30% or less was considered to be a low degree of heterogeneity, 30%–60% to be a moderate degree, and 60% or more to be a high degree. Contributors to heterogeneity were identified using Baujat plot. The publication bias was assessed using the funnel plot including Egger's test.

## 3. Results

### 3.1. Study Selection and Characteristics

In the primary crude analysis, six cohort studies (*n* = 4.362 patients total) reporting HR for OS in high versus low baseline eosinophil groups were eligible. The studies were published between 2016 and 2023 and conducted in the USA, China, Germany, Hungary, France, and in a multinational setting. Reported HR for OS ranged from 0.42 to 2.12, with 95% CIs spanning from 0.01 to 4.23. A total of 4.243 patients were finally included in the multivariable analyses of the six studies (Table 1).

### 3.2. Study Quality According to NOS

Overall, the six cohort studies demonstrate high methodological quality. All achieved strong Selection ratings (3–4★), indicating well-defined, representative cohorts and clear exposure ascertainment. Each study also earned full Comparability (2★), showing that key confounders were consistently controlled. Outcome domains were likewise robust (2–3★), with reliable assessment methods and adequate follow-up—aside from a slight shortfall in follow-up length in Weide et al. [5] (O2 ☆) and a minor lapse in follow-up completeness in Bai et al. [13] (O3 ☆). Taken together, these data attest to rigorous design and execution across the board, with only minimal concerns around the duration and completeness of follow-up in two studies (Table 2).

A study can be awarded a maximum of one star for each numbered item within the Selection and Outcome categories. A maximum of two stars can be given for Comparability: S1: representativeness of the exposed cohort, S2: selection of the nonexposed cohort, S3: ascertainment of exposure, S4: demonstration that outcome of interest was not present at start of study; C: comparability of cohorts on the basis of the design or analysis; O1: assessment of outcome, O2: was follow-up long enough for outcomes to occur, and O3: adequacy of follow up of cohorts; only bold stars count.

### 3.3. Primary Meta-Analysis and Heterogeneity

A random-effects meta-analysis using the DerSimonian–Laird method (log-transformed HR and inverse-variance weighting) yielded a pooled HR of 0.69 (95% CI: 0.36–1.30), suggesting a nonsignificant survival benefit for patients with high baseline eosinophils. However, there was substantial heterogeneity across the six studies (*p* < 0.001; *I*^2^ = 77.8%), indicating that variability in effect size exceeded what would be expected by chance alone.

### 3.4. Outlier Detection and Exclusion Method

**Figure 2:**
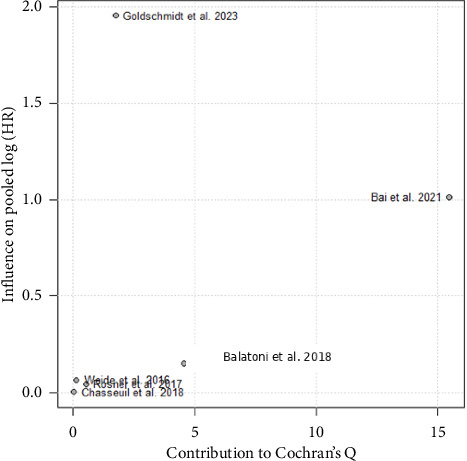
Baujat plot of study influence on heterogeneity and pooled effect (all studies included). Each point corresponds to one cohort study and is positioned according to its contribution to Cochran's Q statistic (*x*-axis, reflecting heterogeneity) and its influence on the overall pooled log-hazard ratio (*y*-axis). Studies in the upper-right quadrant both drive between-study heterogeneity and exert the greatest impact on the pooled estimate. In this plot, Bai et al. [13] is the clear outlier, contributing most to Q and producing the largest shift in the pooled effect, thereby justifying its exclusion from the final meta-analysis.

Baujat plot was constructed to quantify each study's contribution to overall heterogeneity (Cochran's Q) and to the pooled treatment effect in the six‐study meta‐analysis (Figure 2). On this plot, each point represents one cohort, with its horizontal position reflecting the study's contribution to Q (heterogeneity) and its vertical position reflecting the change in the pooled log-HR when the study is omitted. Bai et al. [13] appears furthest to the right and highest, indicating that it contributes disproportionately to between‐study heterogeneity and exerts the largest influence on the pooled estimate. No other study occupies a comparably extreme position. This visualization corroborates the leave-one-out sensitivity analysis and justifies exclusion of Bai et al. [13] from the final random‐effects meta-analysis.

To identify the primary source of heterogeneity, we also performed a leave-one-out sensitivity analysis. Iteratively omitting each study in turn and recalculating *I*^2^ revealed that exclusion of Bai et al. [13] reduced between-study heterogeneity from 77.8% to 33.3% (*p*=0.20). No other single-study omission produced a comparable reduction. Based on this finding, Bai et al. [13] were predefined as an outlier and excluded from the final pooled analysis.

### 3.5. Pooled Effect Size After Outlier Exclusion

**Figure 3:**
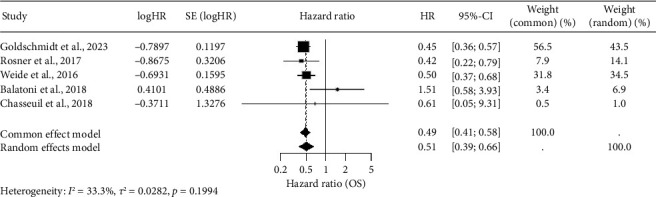
Showing a forest plot of overall survival (OS) by baseline eosinophil status prior to immune checkpoint inhibition. Individual studies comparing high versus low baseline eosinophil counts are displayed with their hazard ratios (HRs) and 95% confidence intervals (CI); square markers indicate the point estimates (log‐scaled) and horizontal lines the CIs. The size of each square is proportional to the inverse‐variance weight in the random‐effects meta‐analysis. The vertical dashed line at HR = 1 denotes no difference in OS between groups. A DerSimonian–Laird random‐effects model (inverse‐variance weighting on log‐transformed HR) was used to pool data from five cohorts, following exclusion of Bai et al. [13] as an outlier identified by Baujat plotting and leave‐one‐out sensitivity analysis. The pooled HR (diamond) is 0.51 (95% CI: 0.39–0.66), indicating a 49% reduction in the hazard of death for patients with high baseline eosinophil counts. Notably, between‐study heterogeneity after exclusion was moderate and not statistically significant (*Q*_4_ = 6.00, *p*=0.20; *I*^2^ = 33.3%).

The meta-analysis was then repeated on the remaining five studies (*n* = 4562). The DerSimonian–Laird random-effects model demonstrated a significant association between high baseline eosinophil counts and improved OS: random effects model HR = 0.51, 95% CI:  0.39–0.66, and *p* < 0.001 (Figure 3).

**Figure 4:**
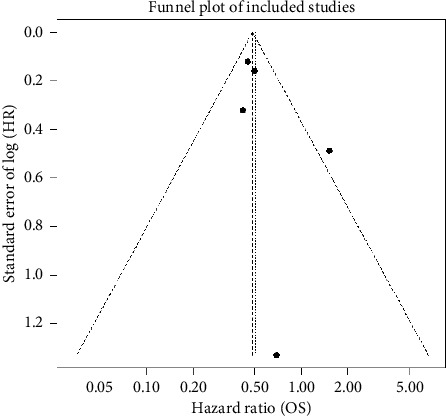
Funnel plot of log‐hazard ratios (HRs) for overall survival by baseline eosinophil status versus their standard errors in five immunotherapy cohorts. Each point represents one study; the vertical dashed line indicates the pooled random‐effects HR on the log scale, and the sloping lines denote the pseudo-95% confidence limits under no bias. The approximate symmetry of the plot and a nonsignificant Egger's test (*p*=0.27) support absence of significant publication bias.

To assess potential small‐study effects and publication bias in the final meta‐analysis (*n* = 5 cohorts: Goldschmid et al. [7]; Rosner et al. [18]; Weide et al. [5]; Balatoni et al. [14]; and Chasseuil et al. [25]), we generated a funnel plot of the log-HRs against their standard errors and performed Egger's regression test. The funnel plot (Figure 4) appeared approximately symmetric around the pooled effect estimate, with no marked under-representation of small studies on either side of the mean. Egger's linear‐regression test yielded a nonsignificant intercept (*p* = 0.27), indicating no statistical evidence of small‐study effects or publication bias.

## 4. Discussion

Eosinophils are increasingly recognized as active orchestrators of antitumor immunity, and several mechanistic studies help explain our finding of improved OS under ICI in patients with high baseline eosinophils. First, eosinophils can directly present antigens and “license” dendritic cells (DCs), thereby enhancing T cell priming. In vitro and in vivo models show that eosinophil‐derived major basic protein and eosinophil peroxidase facilitate antigen uptake by DCs and upregulate costimulatory molecules (CD80/86), promoting both CD4^+^ and CD8^+^ T cell activation [26]. Second, tumor‐homing eosinophils secrete chemokines such as CCL5, CXCL9, and CXCL10 that create chemotactic gradients for cytotoxic T lymphocytes. Carretero et al. [27] demonstrated in murine melanoma models that activated eosinophils normalize the tumor vasculature and release these chemokines, resulting in increased CD8^+^ T cell infiltration and effective tumor rejection. Third, eosinophil‐derived cytokines, including IL-4, IL-5, GM-CSF, and IL-33, shape a more immunogenic microenvironment by supporting DC maturation, augmenting antigen presentation, and polarizing macrophages toward an M1‐like phenotype [28]. Collectively, these effects, including enhanced antigen presentation, directed T cell recruitment, and increased tumor immunogenicity, provide a strong biological rationale for why patients with higher baseline eosinophil counts may experience better outcomes under ICI.

Hence, our meta‐analysis demonstrates that elevated baseline eosinophil counts are robustly associated with improved OS under immune checkpoint therapy: after removal of the Bai et al. [13] outlier, the pooled random‐effects HR was 0.51 (95% CI: 0.39–0.66, *p* < 0.001), indicating a nearly 50% reduction in the risk of death for patients with high versus low eosinophils. The leave‐one‐out and Baujat diagnostics confirmed that the Bai et al. [13] cohort was driving heterogeneity (*I*^2^ 77.8% ⟶ 33.3% after exclusion), and we found no evidence of publication bias (Egger's *p*=0.27). These findings align with aforementioned preclinical and translational data, suggesting that eosinophils may enhance antigen presentation, T cell recruitment, and tumor immunogenicity.

Elevated baseline eosinophil counts may reflect underlying atopic or inflammatory comorbidities, as eosinophils are central effector cells in allergic disorders (e.g., asthma and atopic dermatitis) and helminth infections. Epidemiological studies have reported an inverse association between IgE-mediated atopy and certain cancers, possibly mediated by eosinophilia in allergic individuals [29]. Likewise, paraneoplastic eosinophilia can arise in solid and hematologic malignancies via tumor-derived cytokines (notably interleukin-5), driving recruitment and expansion of eosinophils in peripheral blood [30]. Under ICI therapy, both relative and absolute eosinophil counts often increase early after treatment initiation. In NSCLC patients treated with PD-1/PD-L1 inhibitors, an increase in relative eosinophil count (REC) at 4 weeks (from ∼1.8% to 4.4%) was independently associated with superior disease control and OS (*p* < 0.001) [31]. Similarly, in metastatic renal cell carcinoma, high absolute eosinophil counts (AECs ≥ 329 cells/μL) at four weeks correlated with higher response rates and longer PFS and OS (*p* ≤ 0.03) [32]. Persistent early increases in both AEC and REC have been proposed as dynamic predictive biomarkers in multiple tumor types [33]. AEC and REC convey complementary information: AEC directly measures effector cell burden, whereas REC accounts for shifts in the overall leukocyte composition. Moreover, some studies suggest AEC thresholds may be more robust for predicting immune-related adverse events and treatment efficacy [34], while REC may better capture early dynamic changes in the host immune response [35]. Future work should standardize which metric (or combination thereof) most reliably stratifies patients for ICI treatment.

However, several limitations warrant caution. All included studies were observational, raising the possibility of residual confounding (e.g., concurrent treatments or comorbidities affecting eosinophil levels). The total number of some cohorts is modest, and thresholds for “high” eosinophils varied across studies. Moreover, although heterogeneity fell to moderate levels after excluding Bai et al. [13], unexplained variability remains. Future prospective trials should standardize eosinophil cutoffs, collect comprehensive covariate data, and explore mechanistic correlates (e.g., tumor‐infiltrating eosinophils and cytokine profiles). If confirmed, baseline eosinophil count could serve as a readily available biomarker to stratify patients and personalize immunotherapy strategies.

## 5. Conclusions

Our systematic review and meta‐analysis of five CM cohorts demonstrates that elevated baseline eosinophil counts are significantly associated with improved OS under ICI (pooled HR: 0.51, 95% CI: 0.39–0.66, and *p* < 0.001), after exclusion of an outlier study to reduce heterogeneity (*I*^2^ from 77.8% to 33.3%). Mechanistic evidence supports a role for eosinophils in enhancing antigen presentation, directing CD8^+^ T-cell recruitment via chemokine secretion, and promoting a pro-inflammatory tumor microenvironment. These findings identify baseline eosinophil count as a promising, readily available biomarker for CM patient stratification in immunotherapy. Prospective validation, using standardized eosinophil cutoffs and integrating functional immunologic correlates, will be essential to translate this biomarker into clinical practice.

## Figures and Tables

**Table 1:** Baseline eosinophils predicting overall survival (OS) in melanoma patients undergoing ICI therapy (included in multivariable analysis).

Study (year, location)	Design	*N*	Initial therapy	Eosinophil cutoff	HR (95% CI) for OS	*p* value
Goldschmidt et al. (2023, USA)	Retrosp. multicenter observational cohort study	3314	Anti-PD-1 (*n* = 2144) Anti-CTLA4 (*n* = 308) Anti-PD1 + anti-CTLA4 (*n* = 736) Nonstandard regimen (*n* = 126)	≥ 0.29 ∗ 10^9^/L (vs. < 0.07)	0.454 (0.359–0.574)	< 0.0001
Bai et al. (2021, China)	Ad-hoc analysis, pooled data of two prosp. trials	85	Anti-PD1	> 0.09 ∗ 10^9^/L (vs. ≤ 0.09)	2.123 (1.065–4.233)	0.03
Balatoni et al. (2018, Hungary)	Retrosp. monocenter observational cohort study	47	Anti-CTLA4	> 0.1 ∗ 10^9^/L (vs. ≤ 0.1)	1.507 (0.578–3.924)	0.40
Chasseuil et al. (2018, France)	Retrosp. monocenter observational cohort study	76	Anti-PD1	N/A (“elevated eosinophil count”)	0.69 (0.01–182)	0.89
Rosner et al. (2017, USA)	Retrosp. monocenter observational cohort study	209	Anti-PD1 + anti-CTLA4	> 1.1% (vs. ≤ 1.1)	0.420 (0.224–0.787)	0.007
Weide et al. (2016, Germany)	Retrosp. multicenter observational cohort study	512	Anti-PD1	≥ 1.5% (vs. < 1.5)	0.500 (0.357–0.667)	< 0.001

*Note:* Retrosp. = retrospective, prosp. = prospective.

**Table 2:** The overall quality and risk of bias in the identified studies were evaluated using the NOS (Newcastle–Ottawa quality assessment scale) for nonrandomized controlled trials selected in the present systematic review according to PRISMA.

Study (year, location)	Selection				Comparability	Outcome		
	S1	S2	S3	S4	C	O1	O2	O3
Goldschmid et al. (2023, USA)	★	☆	★	★	★★	★	★	★
Bai et al. (2021, China)	★	☆	★	★	★★	★	★	☆
Balatoni et al. (2018, Hungary)	★	☆	★	★	★★	★	★	★
Chasseuil et al. (2018, France)	★	☆	★	★	★★	★	★	★
Rosner et al. (2017, USA)	★	☆	★	★	★★	★	★	★
Weide et al. (2016, Germany)	★	☆	★	★	★★	★	☆	★

## Data Availability

Derived data supporting the findings of this study are available from the corresponding author upon reasonable request.

## Supporting Information

Supporting Information 1 Table 1 Supporting. PRISMA 2020 checklist. Page MJ, McKenzie JE, Bossuyt PM, Boutron I, Hoffmann TC, Mulrow CD, et al. The PRISMA 2020 statement: an updated guideline for reporting systematic reviews. BMJ 2021; 372:n71. doi: 10.1136/bmj.n71.

Supporting Information 2 Table 2 Supporting. Detailed search strategies for the present meta-analysis.

Supporting Information 3 Table 3 Supporting. Selected studies also reporting progression-free survival (PFS).
