# Supplementary material for: Prognostic Potential of Baseline Eosinophils at the Initiation of Immune Checkpoint Inhibitor Treatment of Metastatic Melanoma: A Systematic Review and Meta-Analysis
Source: J Skin Cancer. 2025 Nov 30;2025:2561307. doi: 10.1155/jskc/2561307 (PMC12682457; doi:10.1155/jskc/2561307)
Supplement: Supporting Information 2 — Table 2 Supporting. Detailed search strategies for the present meta-analysis. [file 2561307.f2.docx]

| ***Table 2suppl:*** *Search strategies in detail* | | | | |
| --- | --- | --- | --- | --- |
| **Datenbank** | **Search date** | **Period** | **Search string** | **Results (n)** |
| PubMed | 31.03.25 | from 2011 | ("Eosinophils"[MeSH Terms] OR eosinophils OR "eosinophil count" OR "eosinophil granulocytes") AND ("Melanoma"[MeSH Terms] OR melanoma OR "malignant melanoma") AND (("immune checkpoint inhibitors" OR "checkpoint blockade" OR "PD-1 blockade" OR "CTLA-4 blockade" OR anti-PD-1 OR anti-PD-L1 OR anti-CTLA-4 OR nivolumab OR pembrolizumab OR ipilimumab OR atezolizumab OR durvalumab OR cemiplimab) OR ("Immunotherapy"[MeSH Terms] OR immunotherapy)) AND ("biomarker" OR "biomarkers" OR "tumor marker" OR "tumor markers" OR "predictive" OR "prognostic" OR "treatment response") | 58 |
| Cochrane Library | 31.03.25 | from 2011 | (melanoma OR malignant melanoma)  AND  (eosinophils OR eosinophil granulocytes)  AND ((checkpoint inhibitors OR PD-1 inhibitors OR CTLA-4 inhibitors) OR immunotherapy)  AND  (biomarkers OR tumor markers OR predictive markers) | 3 |
| Scopus | 17.04.25 | from 2011 | ( TITLE-ABS-KEY ( eosinophils OR "eosinophil count" OR "eosinophil granulocytes" ) )  AND  ( TITLE-ABS-KEY ( melanoma OR "malignant melanoma" ) )  AND  ( TITLE-ABS-KEY ( "immune checkpoint inhibitors" OR "checkpoint blockade" OR "PD-1 blockade" OR "CTLA-4 blockade" OR anti-pd-1 OR anti-pd-l1 OR anti-ctla-4 OR nivolumab OR pembrolizumab OR ipilimumab OR atezolizumab OR durvalumab OR cemiplimab OR immunotherapy ) )  AND  ( TITLE-ABS-KEY ( biomarker OR biomarkers OR "tumor marker" OR "tumor markers" OR predictive OR prognostic OR "treatment response" ) ) | 154 |
