# Supplementary material for: Prognostic Potential of Baseline Eosinophils at the Initiation of Immune Checkpoint Inhibitor Treatment of Metastatic Melanoma: A Systematic Review and Meta-Analysis
Source: J Skin Cancer. 2025 Nov 30;2025:2561307. doi: 10.1155/jskc/2561307 (PMC12682457; doi:10.1155/jskc/2561307)
Supplement: Supporting Information 3 — Table 3 Supporting. Selected studies also reporting progression-free survival (PFS). [file 2561307.f3.docx]

**Tab. 3suppl.** Selected studies also reporting progression-free survival (PFS).

| **Study**  **(Year, Location)** | **Design** | **N** | **Initial therapy** | Eosinophil cut-off | HR  (95% CI)  for OS | p-value | Outcome | Typ of Analysis |
| --- | --- | --- | --- | --- | --- | --- | --- | --- |
| Goldschmidt et al.  (2023, USA) | retrosp. multicenter  observational cohort study | 3314 | anti-PD-1 (n=2144)  anti-CTLA4 (n=308)  anti-PD1+anti-CTLA4 (n=736) Non-standard regimen (n=126) | ≥ 0.29 *10⁹/L  (vs. <0.07) | 0.454  (0.359 - 0.574 | < 0.0001 | OS | multivariate |
| Bai et al.  (2021, China) | ad-hoc analysis, pooled data of two prosp. trials | 85 | anti-PD1 | > 0.09*10⁹/L  (vs. ≤0.09) | 2.123  (1.065 - 4.233) | 0.03 | OS | multivariate |
| Bai et al.  (2021, China) | ad-hoc analysis, pooled data of two prosp. trials | 85 | anti-PD1 | > 0.09*10⁹/L  (vs. ≤0.09) | 1.365  (0.262-7.124) | 0.71 | PFS | univariate |
| Balatoni et al.  (2018, Hungary) | retrosp. monocenter  observational cohort study | 47 | anti-CTLA4 | > 0.1 *10⁹/L  (vs. ≤ 0.1) | 1.507  (0.578 - 3.924) | 0.40 | OS | multivariate |
| Balatoni et al.  (2018, Hungary) | retrosp. monocenter  observational cohort study | 47 | anti-CTLA4 | > 0.1 *10⁹/L  (vs. ≤ 0.1) | 1.909  (1.010-3.609) | 0.0238 | PFS | univariate |
| Chasseuil et al.  (2018, France) | retrosp. monocenter  observational cohort study | 76 | anti-PD1 | N/A ("elevated eosinophil count" | 0.69  (0.01-182) | 0.89 | OS | multivariate |
| Chasseuil et al.  (2018, France) | retrosp. monocenter  observational cohort study | 76 | anti-PD1 | N/A ("elevated eosinophil count" | 0.14  (0.01-4.99) | 0.27 | PFS | multivariate |
| Rosner et al.  ( 2017, USA) | retrosp. monocenter  observational cohort study | 209 | anti-PD1+anti-CTLA4 | >1.1%  (vs. ≤1.1) | 0.420  (0.224 - 0.787) | 0.007 | OS | multivariate |
| Weide et al.  (2016, Germany) | retrosp. multicenter  observational cohort study | 512 | anti-PD1 | ≥ 1.5%  (vs. < 1.5) | 0.500  (0.357 - 0.667) | < 0.001 | OS | multivariate |

Retrosp. = retrospective, prosp. = prospective
